# The beginning of a new era: pioneering direct screens for RNA modulators

**DOI:** 10.1038/s41392-022-01069-w

**Published:** 2022-07-21

**Authors:** F. X. Reymond Sutandy, Rebecca George Tharyan, Christian Münch

**Affiliations:** 1 Institute of Biochemistry II, Goethe University Frankfurt, Faculty of Medicine, Frankfurt am Main, Germany; 2 Frankfurt Cancer Institute, Frankfurt am Main, Germany; 3 Cardio-Pulmonary Institute, Frankfurt am Main, Germany

**Keywords:** Drug discovery, Genetics

In a recent study published in *Nature,*^1^ Aguilar et al. describe the small molecule X1 that targets the long non-coding RNA *Xist*. Upon X1 binding, *Xist* changes its structure, which prevents its role in X-chromosome inactivation (XCI).

The vast majority of small molecule screens in drug discovery focus on targeting proteins.^2,3^ However, protein coding sequences merely constitute ~1.5% of the human genome and only a small fraction of these proteins are currently targetable.^2^ In contrast, 90% of the human genome is transcribed into RNA in different forms, which makes RNAs compelling targets for small molecule drugs.^3^ Modulating mRNAs with small molecules could be an alternative route to change the levels of the corresponding protein products and their activities, especially for currently un-druggable proteins. Considering the diverse functions carried out by different classes of RNAs in cells, altering RNAs would provide a broad range of potential drug targets in the human genome. While modulating RNAs with small molecules carries a number of advantages, identifying RNA-targeting small molecules has proven difficult; previous compounds had been identified indirectly by phenotypic screens.^1–3^ Systematic approaches to screen for RNA-targeting compounds had been lacking.

**Fig. 1:**
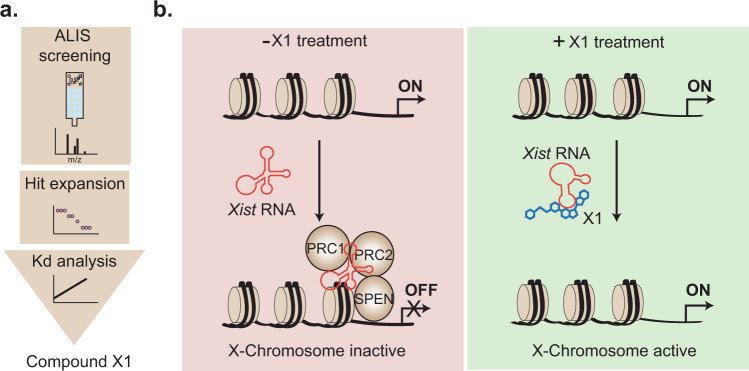
An ALIS-based RNA-binding screen revealed compound X1 as a small molecule that specifically targets *Xist* RNA and modulates its activity. **a** ALIS screening was performed targeting the RepA domain of *Xist* RNA. Compound X22 was identified in the screen and further validated. Hit expansion studies with X22 were carried out and resulting affinities evaluated and ranked. Ultimately, Kd analyses were used to validate several hits, including the original hit X22, and its analog compound X1. Compound X1 yielded the highest affinity to RepA and was used to perform functional assays to modulate *Xist* RNA both in vitro and in cells. **b** The Xist RNA recruits PRC1, PRC2 and SPEN to inactivate the X chromosome by epigenetic modification (left). X1 disrupts the *Xist* RNA structure upon binding and compromises *Xist* RNA-protein interactions, resulting in pertained X chromosome activation (right).

To address this pertaining gap, Aguilar and colleagues leveraged the automated ligand identification system (ALIS), and performed a large-scale unbiased compound screen for the 431 nt-long GC-rich RepA domain of the non-coding RNA *Xist* (Fig. 1a). *Xist* is the main regulator of XCI, which orchestrates epigenetic changes that spread along the entire X chromosome to trigger inactivation of its genes.^4,5^ Several domains on *Xist* are responsible for gene silencing during XCI initiation, among them is RepA.^4^ The ALIS-based screen yielded X22 as a single positive hit from 50,000 tested compounds. A secondary analog screen with 20 compounds expanded from X22 identified compound X1 as the highest affinity binder to RepA. X1 exhibited drug-like properties, which makes it a suitable candidate for small molecule targeting *Xist* RNA. Strikingly, unlike RNA-binding small molecules identified earlier, X1 displayed high selectivity to RepA, even when compared to RNAs with similar size.

RepA facilitates *Xist* spreading during XCI via its interactions with epigenetic regulators, including polycomb repressive complex 2 (PRC2) and the RNA recognition motif of SPEN.^4^ In vitro interaction assays indicated that compound X1 selectively competed for binding of RepA to both PRC2 and SPEN with varying inhibitory effects. RNA immunoprecipitation (RIP) studies on mouse embryonic stem cells (ESCs) confirmed that X1 maintained its inhibitory properties in cells and selectively disrupted PRC2 and SPEN interactions with *Xist*. Strikingly, the RIP assay in ESCs also recapitulated the differences in PRC2 and SPEN inhibition by X1 observed in vitro. Additionally, deleting RepA in fibroblasts entirely abrogated the inhibitory effects of X1, validating that X1 effects were mediated by RepA in cells.

Ultimately, Aguilar and colleagues tested whether X1 interaction with the *Xist* RepA domain had a functional impact on XCI. They observed defects on embryoid body outgrowth of female ESCs upon exposure to compound X1. This effect is *Xist-*dependent as XY male and XO female cells did not show the growth defects. XCI is characterized by PRC1-mediated monoubiquitylation of lysine 119 on histone H2A (H2AK119ub), followed by enrichment of repressive histone marks, such as tri-methylation of lysine 27 on histone 3 (H3K27me3), via PRC2 activity on inactive X chromosomes (Xi).^5^ Immunostaining assays confirmed enrichment of those epigenetic markers on Xi, which were significantly abolished by treatment with X1 without affecting *Xist* expression itself. CHIP-seq analyses showed that the inhibition of PRC2 and H3K27me3 accumulation observed upon X1 treatment were specific to Xi and not observed on active X chromosome (Xa) or autosome. In addition, allele-specific RNA-sequencing revealed that X1 administration prompted increased expression of Xi genes with only minor autosomal effects, suggesting minimal X1 off-target effects to modulate XCI. Notably, this X1 effect is reversible as retrieving X1 treatment restored embryoid body growth and Xi silencing.

To investigate further the molecular mechanistic of X1 inhibition, Aguilar et al. analyzed RepA with and without X1 by small-angle-X-ray scattering. Overall, these analyses showed minor changes in RepA size and shape distribution and suggested that RepA underwent conformational changes upon interaction with X1 without a significant loss of its RNA structure. Further modeling of the RepA 3D structure revealed a distinct RepA structure and reduced conformational heterogeneity upon X1 addition, when compared to its free form. Aguilar et al. proposed that this X1-induced RepA structure reduces RepA affinity for its associated protein interactors, leading to X chromosome activation (Fig. 1b).

Altogether, the described work pioneers and exemplifies a direct RNA-binding screen design to identify small molecules that bind to RNA with the potential to exhibit phenotypic effects. In light of the recent increases in efforts to screen for RNA binding small molecules, this work provides a unique blueprint for such a screen. Adjustment to the ALIS design, which was originally developed to target proteins, could improve the identification of RNA-binding molecules and maximize the benefit to attain possibly higher output of identified small molecules in the future than currently presented in this study. In regard to ligandability, *Xist* RNA is an untypical target with its large size and high likelihood of being mostly unstructured. However, this work has demonstrated that the ALIS approach can overcome this challenge and could potentially be extended to any type of RNA, especially when structure information is not available. It would still be intriguing to evaluate the versatility of this design to target RNA with different physical properties, for instance small or highly structured RNAs. This work highlights an exciting possibility to utilize RNA-targeting compound to modulate currently undruggable proteins and cellular pathways beyond their protein-driven functions.
